# Methemoglobinemia Preceded by Food Poisoning: A Case Report

**DOI:** 10.7759/cureus.73412

**Published:** 2024-11-10

**Authors:** Abdulrahman Tabrah

**Affiliations:** 1 Internal Medicine, Al-Furat General Hospital, Baghdad, IRQ

**Keywords:** ascorbic acid, food poisoning, methemoglobinemia, methylene blue, oxygen delivery

## Abstract

This case report explores the presentation and management of methemoglobinemia, a condition characterized by elevated levels of methemoglobin in the blood, leading to reduced oxygen delivery to tissues. We present a case of a patient who developed methemoglobinemia following food poisoning, highlighting the clinical features observed during admission, such as cyanosis, dyspnea, and altered mental status. The patient was treated with methylene blue and ascorbic acid, which effectively reduced methemoglobin levels. This report emphasizes the importance of recognizing methemoglobinemia in patients with a history of food poisoning and the need for prompt diagnosis and treatment to improve patient outcomes. We discuss the underlying mechanisms, potential complications, and the significance of individualized treatment approaches in managing this condition.

## Introduction

Methemoglobinemia is a blood disorder where the ferric form of hemoglobin appears in the blood. While this form can carry oxygen, it struggles to release it effectively, leading to a range of uncomfortable symptoms such as headaches, dizziness, nausea, and cyanosis (a bluish tint to the skin). This condition is rarely inherited and is more commonly caused by exposure to certain oxidizing agents, like local anesthetics and quinolone antibiotics.

Under normal circumstances, oxygen binds to hemoglobin when it’s in the ferrous state (Fe^2+^). However, in methemoglobinemia, the heme iron shifts from ferrous (Fe^2+^) to ferric (Fe^3+^), preventing it from binding oxygen. As a result, the normal ferrous heme becomes overly eager to hold onto oxygen, causing a leftward shift in the oxygen dissociation curve. This shift ultimately leads to functional anemia, as the blood’s ability to carry oxygen decreases [1].

Methemoglobinemia can be triggered by various medications and environmental factors, and it may present similarly to other respiratory issues, such as exacerbations of chronic obstructive pulmonary disease (COPD). While congenital methemoglobinemia due to a deficiency in cytochrome b5 reductase is quite rare, its exact incidence is not well-known. Some populations, such as the Siberian Yakuts, Athabaskans, Eskimos, and Navajo, have been found to have a higher prevalence of this condition [1,2].

## Case presentation

**Figure 1:**
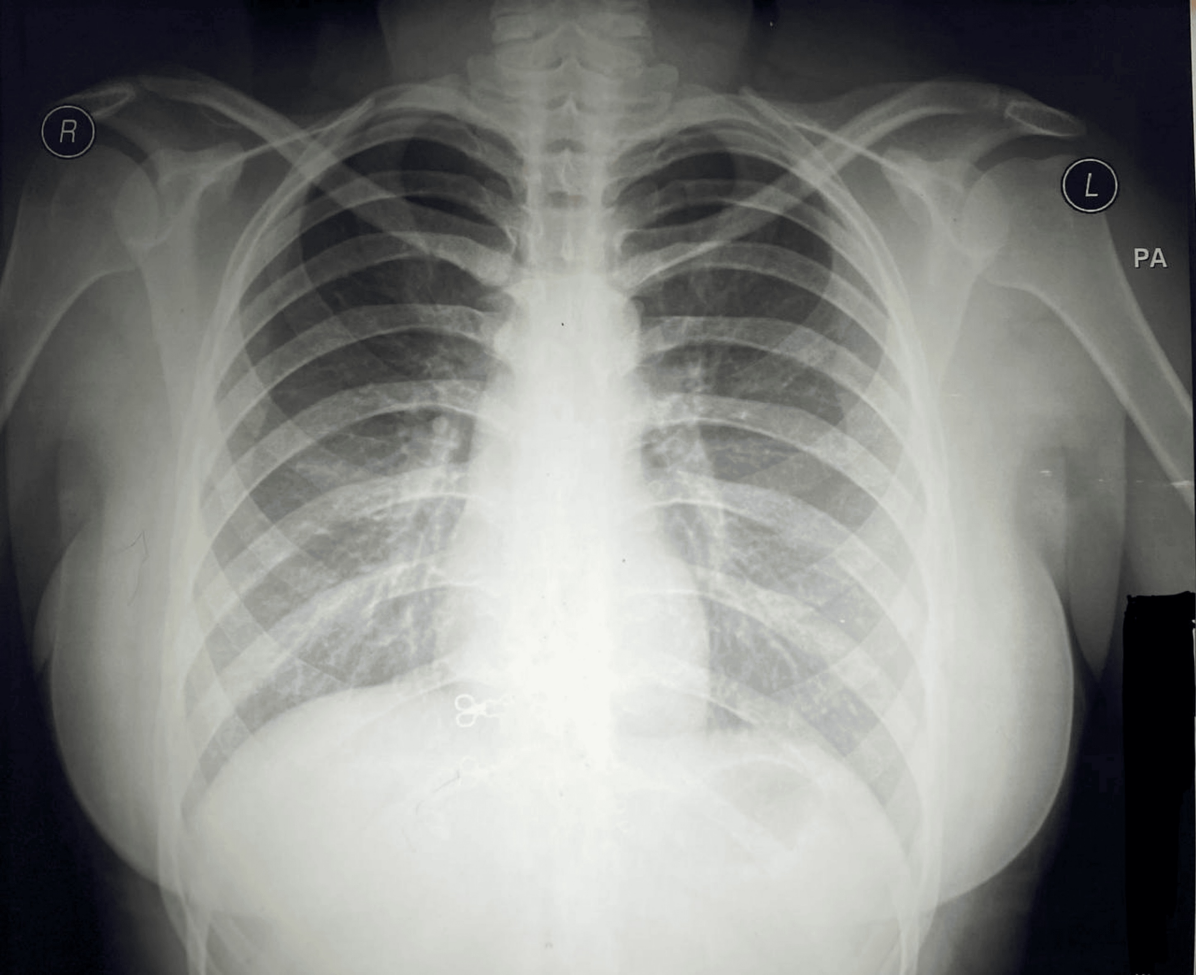
Chest X-Ray

A 52-year-old female presented to the emergency department with shortness of breath (SOB) and cyanosis for one day. She had two days of vomiting and mild fever due to food poisoning, for which she had been receiving supportive treatment and she didn't receive any antibiotics. On examination, she was alert and oriented. Her abdominal exam was unremarkable, and her chest examination revealed good air entry bilaterally with normal percussion and normal X-ray (Figure 1). Her vitals included a blood pressure of 130/100 mmHg, heart rate of 95 bpm, and oxygen saturation of 85% on room air. Despite administering oxygen, her saturation did not improve. 

Initial investigations, including complete blood count and renal function tests, were normal. An arterial blood gas (ABG) showed normal pO_2_ but significantly elevated methemoglobin (FMetHb) levels at 16.8%. The patient also had hypokalemia (Table 1).

**Table 1:** Lab findings FMetHb: methemoglobin

Test	Patient result	Normal range
Serum creatinine	0.7mg/dL	0.6-1.2 mg/dL
Urea	3.5 mmol/L	2.5-7.5 mmol/L
Hemoglobin	13.9 g/dL	11.5-16.5 g/dL
White blood cells	7,000/µL	4,000-11,000/µL
Platelets	180,000/µL	150,000-450,000/µL
Sodium	134 mmol/L	135-145 mmol/L
Potassium	2.5 mmol/L	3.5-5.0 mmol/L
Chloride	105 mmol/L	98-106 mmol/L
Bicarbonate	23.2 mmol/L	22-26 mmol/L
pCO_2_	31.1 mmHg	35-45 mmHg
pO_2_	90.7 mmHg	80-100 mmHg
FMetHb	16.8 %	0.0-1.5 %

The patient was admitted to the ward and received methylene blue as part of the treatment regimen, along with potassium chloride (KCl) for electrolyte management.

Methylene blue is a key treatment option, often used alone or in combination with ascorbic acid. It is typically administered orally at doses ranging from 100 to 300 mg per day, tailored according to the patient’s methemoglobin (Met-Hb) levels. However, its effectiveness can be limited in patients with specific hemoglobin variants, such as Hb Cheverly and Hb Evans, which may not respond adequately to the medication.

## Discussion

Methemoglobinemia is a rare but potentially life-threatening condition that results from the oxidation of hemoglobin from its ferrous (Fe^2+^) to ferric (Fe^3+^) state, diminishing its ability to carry oxygen effectively [1]. It presents clinically with symptoms like cyanosis and hypoxemia that are resistant to oxygen therapy, as was seen in this case where the patient’s oxygen saturation did not improve despite supplemental oxygen [2].

The management of methemoglobinemia requires prompt identification and treatment to avoid severe complications such as tissue hypoxia, which can become fatal when methemoglobin levels exceed 35% [2]. Methylene blue, the first-line treatment, helps by accelerating the conversion of methemoglobin to hemoglobin. In this case, the administration of methylene blue resulted in a swift clinical improvement, demonstrating its efficacy [3].

Additionally, ascorbic acid is sometimes used alongside methylene blue as adjunctive therapy, especially in cases where methemoglobin levels are significantly elevated or treatment response is delayed [3]. This combined approach facilitates faster reduction of methemoglobin levels, as ascorbic acid aids in the reduction of methemoglobin [3]. In severe cases of ineffective methylene blue, alternatives like exchange transfusion or hyperbaric oxygen therapy may be considered [3].

This case further underscores the importance of identifying the causative agent. Here, the patient’s condition was linked to food poisoning, which likely introduced oxidizing agents that triggered the methemoglobinemia [2]. A thorough history and identifying potential oxidant exposures are crucial for effectively managing this condition [3]. Screening for glucose-6-phosphate dehydrogenase (G6PD) deficiency is also essential since methylene blue is contraindicated in these patients due to the risk of hemolysis [3].

The patient’s food poisoning likely introduced an oxidizing agent, which triggered methemoglobin formation. Similar to the case of the 41-day-old infant who developed methemoglobinemia after consuming nitrite-containing well water, this patient’s condition was likely multifactorial [4]. The combination of infection and exposure to an oxidizing agent, possibly from contaminated food, created an environment where methemoglobinemia could develop.

Early recognition and treatment of methemoglobinemia are essential to avoid severe complications, such as tissue hypoxia and potential cardiopulmonary failure. Methylene blue, the first-line treatment, is critical in reversing the oxidized hemoglobin back to its functional form. In the case of this 52-year-old female, prompt administration of methylene blue would have been expected to rapidly improve her clinical condition, just as it did in the previously discussed infant case. Additionally, this case reinforces the importance of identifying potential environmental or dietary causes of methemoglobinemia, such as nitrite-containing food or water, to prevent recurrence. While congenital methemoglobinemia is rare, acquired forms can result from various exposures, making a thorough history essential in both diagnosis and prevention.

## Conclusions

In conclusion, this case underscores the critical importance of prompt recognition and treatment of methemoglobinemia, especially in the context of environmental or dietary exposure to oxidizing agents, such as those associated with food poisoning. The patient's resistance to oxygen therapy and rapid improvement following methylene blue administration emphasize the effectiveness of early intervention in preventing severe complications like tissue hypoxia and cardiopulmonary failure. Furthermore, this case highlights the value of a thorough patient history in identifying potential sources of oxidizing agents, mirroring the recurrence of methemoglobinemia seen in the infant case linked to nitrite-containing well water. Prophylactic measures, particularly in at-risk populations, could help prevent future episodes, and the timely administration of first-line treatments remains essential in ensuring positive patient outcomes.
